# Rethinking Health Care Regulatory Sandboxes Under the European Union AI Act

**DOI:** 10.2196/98653

**Published:** 2026-10-30

**Authors:** María Flores Ceballos, Fernando Cagigas Villar, Alberto López Tejerina

**Affiliations:** 1 Escuela Superior de Ingeniería y Tecnología (ESIT), Universidad Internacional de La Rioja, Avenida de la Paz, 137, Logroño, 26006, Spain,; 2 AIR Institute (Fundación Instituto de Investigación Internacional en Inteligencia Artificial y Ciencias de la Computación), Av. Santiago Madrigal, 39, Salamanca, 37003, Spain

**Keywords:** deep learning, delivery of health care, federated learning, European Union AI Act, EU AI Act, regulatory sandboxes, artificial intelligence, AI

## Abstract

AI models have enabled substantial advances in health care. Since the entry into force of the European Union (EU) AI Act in 2024, these systems have become subject to a common regulatory framework in the EU intended to harmonize AI governance across all member states. One of the act’s distinctive features is that it accommodates existing economic, technological, and regulatory differences among European countries by leaving certain implementation responsibilities to national authorities. This is evident in regulatory sandboxes, supervised environments for developing, testing, and validating AI systems before deployment. In this viewpoint, we provide a policy-oriented analysis of health care regulatory sandboxes under the EU AI Act and propose recommendations for their design. These recommendations address the need to document model compression techniques that may affect the classification of large high-risk systems above the 10^25^ floating point operations per second threshold, incorporate explainability into AI systems from the design stage, identify and document biases in health care datasets, clarify the treatment of federated learning parameter updates under the General Data Protection Regulation, and strengthen the oversight of AI agents in clinical environments. The implementation timeline makes this discussion particularly timely given the approaching deadline of August 2, 2027, by which all member states must have established at least one national regulatory sandbox.

## Introduction

The integration of AI into European health care systems represents a major transformation in contemporary medicine, with significant implications for diagnostic precision, personalized treatment, and care efficiency [1]. These systems are currently regulated in the European Union (EU) under the AI Act, which establishes a common legal framework based on the risk profile of AI systems across all member states. However, the European landscape remains structurally heterogeneous as countries differ substantially in technological capacity, economic investment, and the maturity of their national AI-related regulatory environments. For this reason, although the AI Act provides a harmonized legal architecture, it leaves certain implementation responsibilities in the hands of individual member states, among which regulatory sandboxes occupy a relevant position.

This Viewpoint aims to provide a set of recommendations for the design of regulatory sandboxes in the health care sector, one of the most sensitive domains for the deployment of AI systems. The recommendations are informed by a policy-oriented analysis of selected challenges under the EU AI Act. They address both the governance of sensitive medical datasets and the treatment of recent AI models and techniques whose legal interpretation may prove challenging. Given that by August 2, 2027, each member state must ensure the existence of at least one regulatory sandbox, we argue that this discussion is both timely and potentially useful for the design and operational development of such environments.

## Regulation of AI in the EU Under the AI Act

The EU AI Act, the world’s first comprehensive legal framework for the regulation of AI, entered into force on August 1, 2024 [2]. This milestone represents a pivotal moment in technology governance by establishing harmonized rules for the internal market (Article 1(a)); fostering the development and uptake of human-centric and trustworthy AI systems (Article 1(b)); and ensuring a high level of protection of health, safety, and fundamental rights (Article 1(c)).

The AI Act follows a phased implementation timeline (Table 1). The first provisions began to apply on February 2, 2025, followed by governance rules and obligations for general-purpose AI models on August 2, 2025. Although August 2, 2026, remains the regulation’s general date of application, the requirements applicable to high-risk AI systems under Chapter III, Sections 1 to 3, have been postponed. Following Regulation (EU) 2026/1744, these requirements apply from December 2, 2027, to systems classified as high-risk under Article 6(2) and Annex III, which may include AI systems used in health care–related essential public services, and from August 2, 2028, to systems classified under Article 6(1) and Annex I, including AI systems that constitute or are safety components of regulated products, such as medical devices subject to third-party conformity assessment.

**Table 1.** Milestones in the implementation timeline of the European Union AI Act relevant to health care regulatory sandboxes.

Key date	Implementation milestone
February 2, 2025	The first provisions of the AI Act became applicable, including prohibited AI practices and AI literacy requirements.
August 2, 2025	Governance rules and obligations for general-purpose AI models became applicable.
August 2, 2026	The AI Act reached its general date of application, although specific requirements for high-risk AI systems remain subject to later deadlines.
August 2, 2027	Member states must ensure that at least one national AI regulatory sandbox is operational.
December 2, 2027	Requirements for certain high-risk AI systems become applicable.
August 2, 2028	Requirements for high-risk AI systems embedded in or constituting regulated products become applicable.

The progressive implementation of the regulation constitutes a unique opportunity to identify and address, in a gradual and evidence-based manner, the difficulties that may arise during its application. The key date for this Viewpoint is August 2, 2027, by which member states must ensure that at least one national AI regulatory sandbox is operational. Originally set for August 2, 2026, this deadline was subsequently postponed, reflecting the practical challenges involved in designing regulatory sandboxes across member states.

Although it is frequently argued that the AI Act may hinder innovation, this interpretation reflects a conceptual conflation between research activities and market regulation. The AI Act does not restrict the development of or experimentation with AI systems. Instead, it regulates the conditions under which such systems may be placed on the market or put into service within the EU. Its scope is therefore focused on commercialization and high-risk deployment rather than on upstream scientific or technological innovation.

The AI Act establishes a risk-based classification framework for AI systems. Within this framework, “minimal-risk” systems are subject to very limited regulatory obligations and typically include applications such as ethically designed video games. Second, “limited-risk” systems must comply with specific transparency requirements, including the obligation to inform users when content has been generated or modified using AI. Third, “high-risk” systems are subject to significantly more stringent obligations, encompassing enhanced transparency, human oversight, risk management procedures, and postmarket monitoring, and include, among others, remote biometric identification technologies and certain autonomous vehicle applications. Finally, “prohibited” AI systems are those considered to pose an unacceptable risk to safety, human life, or fundamental rights and are therefore banned from being placed on the market or put into service within the EU.

## Legal Uncertainty and Implementation Challenges of the AI Act in Health Care

The health care sector presents intrinsic characteristics—such as high-stakes clinical decision-making, the systematic processing of sensitive data, and the preservation of clinical autonomy—that complicate the implementation of AI governance. Accordingly, digital health AI systems may fall within the high-risk categories established by the AI Act (Annex III). At the same time, the regulation provides narrowly tailored exceptions for specific health care–related contexts, including those referred to in Recital 29 and Article 5(1)(f). Within this framework, the regulation relies predominantly on conformity assessments conducted by providers, with comparatively limited independent external oversight. Such self-assessment mechanisms may be vulnerable to technical misinterpretation or informational asymmetries. This concern becomes particularly salient when health care professionals or hospitals themselves qualify as AI providers [3,4] given their often limited organizational and technical capacity for AI governance.

Moreover, the AI Act uses a computational threshold of 10^25^ floating point operations per second (FLOPS) as a presumption of high-impact capabilities for general-purpose AI models and, consequently, as a criterion for their classification as models with systemic risk. However, computational scale alone may not fully capture the specific risks associated with health care applications built on such models [5,6].

Regulatory complexity is further amplified by the interaction between the AI Act and the General Data Protection Regulation (GDPR) [7]. In health care, compliance must satisfy AI-specific and data protection requirements. Previous analyses also note limited attention in the AI Act to transparency, international data transfers, and accountability [8-10].

Parallel legislative initiatives such as the AI Liability Directive have been proposed to reinforce patient protection and facilitate access to remedies [3,11]. Complementary proposals include digital information instruments such as the AI Passport [12] intended to enhance transparency and mitigate risks in clinical AI deployment.

The AI Act incorporates concepts whose practical interpretation may remain uncertain, most notably “vulnerability.” Article 5 addresses vulnerability in the context of exploitation based on factors such as age, disability, or economic status but does not provide detailed operational criteria for identifying vulnerable groups. This lack of specificity has attracted academic criticism. Furthermore, the obligation to report serious incidents referred to in Recital 96 may be affected by divergent national interpretations of what constitutes a “public service,” particularly in health care. Such inconsistencies risk undermining uniform application across member states and have been widely discussed in the literature [13-15].

Finally, these structural and conceptual ambiguities intersect with ongoing debates on algorithmic transparency and nondiscrimination in medical AI. The AI Act’s requirement of “appropriate transparency” for high-risk systems remains insufficient in clinical contexts, where explainability is not merely a regulatory formality but a prerequisite for informed medical decision-making [6]. The absence of explicit mandates regarding established interpretability techniques limits the practical enforceability of transparency obligations [14]. Similarly, scholarship has addressed unresolved questions concerning medical liability [16] and the amplification of gender biases in health care AI teams [17]. The regulation’s reliance on broadly framed requirements to implement “appropriate measures” against discrimination lacks the technical granularity necessary to address complex bias patterns in medical datasets, particularly those affecting underrepresented demographic groups.

This section highlights the complexity of health care under the AI Act and the need for a nuanced regulatory approach. As noted in the work by Gilbert [18], no law can foresee all possible scenarios. Therefore, it is a shared responsibility to design adaptive solutions and contribute to the AI Act’s evolution so that its intended objectives can be effectively achieved.

## How the AI Act Combines Harmonization With Member State Autonomy

The AI Act establishes a harmonized regulatory framework across the EU while simultaneously embracing differences across member states. As a European regulation, it is directly applicable in all member states without requiring transposition into national law. This means that, from the date of application, national authorities, companies, health care institutions, and AI developers are legally obliged to comply with its provisions even in the absence of additional national legislation.

The regulation follows a progressive implementation timeline, with different provisions becoming applicable in stages until full applicability is reached. During this transitional period, a regulatory gap may emerge in which not all obligations are yet enforceable. In parallel, supervisory bodies begin to operate and issue guidance to clarify permissible practices, thereby reducing uncertainty and supporting early compliance. At the European level, the AI Office [19] within the European Commission and the European Artificial Intelligence Board coordinate the implementation of the regulation, contributing to the alignment of regulatory practices across member states. More broadly, this design allows the AI Act to evolve through practical experience, consistent with the regulation of emerging technologies, where innovation often outpaces legislation.

Although the AI Act does not require national transposition, member states retain the ability to adopt complementary measures to operationalize its provisions. This occurs for several reasons. First, the regulation requires the designation or creation of competent authorities, supervisory bodies, and market surveillance mechanisms at the national level. Second, member states may define procedural aspects such as inspection regimens, enforcement practices, and administrative sanctions. Third, they may introduce sector-specific rules to adapt the regulation to particular domains such as health care, including provisions related to clinical evaluation, hospital deployment, and the governance of medical data.

Within this framework, regulatory sandboxes play a central role by enabling the supervised testing of AI systems prior to their market deployment, particularly for high-risk applications such as those in health care. Article 57 of the AI Act requires member states to ensure that at least one AI regulatory sandbox is established at the national level and becomes operational before August 2, 2027. The regulation explicitly provides for this instrument to facilitate technological experimentation, enable early risk assessment, and support responsible innovation under public supervision. In practice, a regulatory sandbox allows a company, hospital, or research institution to submit an AI system for testing in a controlled environment authorized by the competent authority, where its performance and its potential impact on safety, fundamental rights, data protection, transparency, and nondiscrimination are assessed.

However, the decentralized implementation of regulatory sandboxes may generate differences in evaluation methodologies, supervisory practices, and access conditions across member states. This may lead to regulatory fragmentation, unequal opportunities for experimentation, and divergent approaches to the evaluation of health care AI systems.

Member states with smaller health systems may therefore face greater difficulties in applying complex testing and documentation requirements. As a result, similar health care AI systems could be evaluated differently across national sandboxes. Rather than pursuing further binding legal harmonization, greater practical convergence could be promoted through common evaluation criteria, transparency standards, and supervisory guidance. Some of the criteria proposed in this Viewpoint are intended to contribute to this convergence in the health care context. European mechanisms such as EU regulatory sandboxes for AI could help translate these proposals into practice by providing shared methodological frameworks, technical guidance, capacity-building measures, and cooperation among national authorities.

## Recommendations for the Design and Implementation of AI Regulatory Sandboxes in Health Care

### Overview

The preceding discussion highlights several regulatory uncertainties that may affect the implementation of AI regulatory sandboxes in health care. Building on these challenges, the following subsections present practical proposals for AI regulatory sandboxes in health care under the EU AI Act, focusing on technical and legal difficulties that may emerge when medical AI systems are tested under regulatory supervision. These recommendations focus on model assessment, explainability, dataset governance, federated learning (FL), and AI agents. They address what, in our view, are among the main emerging regulatory and technical challenges in the design and implementation of health care AI regulatory sandboxes.

### Document Model Compression Methods That May Affect Risk Classification

As previously discussed, the AI Act establishes a computational threshold of 10^25^ FLOPS as a presumption of high-impact capabilities for general-purpose AI models and, consequently, as a criterion for identifying models with systemic risk. FLOPS quantify the amount of computation performed during model training. In general, models with larger numbers of parameters tend to require greater computational resources during training and, consequently, a higher number of FLOPS. In neural network architectures, these parameters typically correspond to the weights and biases learned during training.

However, recent advances in model design have led to the emergence of architectures such as tensor neural networks [20], which enable a substantial reduction in the number of parameters while preserving comparable functional performance. When such compression techniques are incorporated before or during training, they may reduce the computational resources required to train the model. Consequently, an architecture that would otherwise require training compute above the FLOPS threshold may remain below it when trained using these techniques.

For example, a large language model exceeding the 10^25^ FLOPS threshold could be integrated into a hospital information system to summarize patient records. Compression methods could subsequently produce a smaller model that preserves comparable performance for this task while falling below the computational threshold. The model would therefore no longer automatically meet the compute-based presumption of systemic risk even though clinically relevant risks could remain, such as omitting an allergy, medication, or previous diagnosis from a patient summary. A similar situation could arise with compressed multimodal models used to analyze medical images, as well as with other AI-based medical tools that retain clinically significant capabilities despite substantial reductions in computational complexity.

In light of this, we recommend that developers explicitly disclose whether a given model relies on tensor-based architectures or other forms of parameter compression. Where such techniques are used, developers should also report both the original number of parameters and the reduced parameter count after compression. In a health care regulatory sandbox, this information could be considered together with clinically relevant performance to determine whether compression has altered the model’s practical capabilities or risks before deployment.

### Promote Explainability Approaches That Reflect Dataset Characteristics

According to Article 13 of the AI Act, “High-risk AI systems shall be designed and developed in such a way as to ensure that their operation is sufficiently transparent to enable deployers to interpret a system’s output and use it appropriately. An appropriate type and degree of transparency shall be ensured with a view to achieving compliance with the relevant obligations of the provider and deployer” [2]. The regulation does not impose specific technical requirements for explainability, such as the use of commonly cited methods in the literature, including Shapley additive explanations [21] or local interpretable model-agnostic explanations [22]. Instead, it adopts a functional approach centered on ensuring interpretability of outputs in context.

In this regard, we recommend selecting explainability approaches according to the clinical question and the characteristics of the underlying dataset. Local interpretable model-agnostic explanations is primarily used to explain individual predictions (eg, why a model assigns a high risk score to a particular patient), whereas Shapley additive explanations can provide both individual and aggregated feature importance across the patient population (eg, which clinical variables are generally most influential in predicting disease progression).

However, when the dataset is heterogeneous and the aim is to determine whether the relevance of particular variables changes across different regions of the patient population, complementary sensitivity analysis approaches may be more informative (eg, identifying a variable with moderate overall importance that becomes highly influential in a specific subgroup of patients).

The sensitivity analysis method developed in the study by Pizarroso et al [23] illustrates this by revealing variables with moderate average influence that become highly influential in specific regions of the dataset. In its application to Parkinson disease progression, sex showed the highest global importance and a comparatively consistent influence across patients, whereas jitter (absolute) and pitch period entropy became particularly influential in specific patient cohorts, a pattern that the authors suggested could be related to different Parkinson disease subtypes.

### Adopt Inclusive and Bias-Aware Documentation for Health Care Datasets

The AI Act imposes explicit data governance obligations on high-risk AI systems that rely on training data. Under Article 10(1), such systems must be developed based on training, validation, and testing datasets that comply with the quality requirements set out in Articles 10(2) to 10(5). More specifically, Article 10(2)(f) requires an examination of possible biases that may affect health and safety, negatively impact fundamental rights, or lead to unlawful discrimination, while Article 10(2)(g) requires appropriate measures to detect, prevent, and mitigate those biases. In addition, Article 10(3) requires datasets to be relevant; sufficiently representative; and, to the extent possible, free of errors and complete in light of their intended purpose. Although these requirements are especially important in health care, their deliberately general formulation creates uncertainty when medical AI systems are trained on complex or potentially unbalanced datasets.

In this context, we recommend that dataset documentation and auditing explicitly identify potential sources of bias, with particular attention to representation bias in clinically relevant subgroups [24]. Where adequate representation cannot be achieved, this limitation should be clearly justified in the technical documentation as otherwise it may distort downstream clinical decision-making [25]. We also recommend the use of inclusive and precise language in both dataset and model documentation, especially regarding sex and gender variables, to avoid forms of exclusion that may affect the lesbian, gay, bisexual, transgender, and queer community and undermine both representational fairness and clinical validity.

### Classify FL Parameter Updates as Personal Data Under the GDPR

Since its original proposal in 2016 [26], FL has gained increasing relevance in AI development. Its main advantage lies in allowing a model to be trained across local datasets without transferring those datasets between institutions. This is particularly valuable in health care, where data are often large and highly sensitive [27,28]. At the same time, because the local datasets remain inaccessible, assessing potential sources of bias may become more difficult. This limitation raises transparency and explainability concerns and supports the need for documentation on how each local dataset was obtained [29].

However, FL also presents important limitations [30]. In addition to the risk of malicious local modifications that may introduce bias, FL is vulnerable to privacy attacks aimed at inferring information about local datasets from the parameter updates transmitted to the central server. This raises a legal question under Article 4(1) of the GDPR, which defines personal data as “any information relating to an identified or identifiable natural person.” In this context, the relevant issue is whether information contained in local datasets can be reconstructed from trained local models or from the exchanged parameter updates.

In this respect, the TechDispatch report on FL states that there is a risk that part of the personal data used in the training could be extracted from the resulting models. Thus, an assessment should be performed on a case-by-case basis to determine whether models need to be considered as personal data [30]. Similarly, the European Data Protection Board [31] notes that the required documentation should include “information on the technical and organisational measures taken while designing the AI model to reduce the likelihood of identification, including the threat model and risk assessments on which these measures are based,” as well as “the documentation demonstrating the AI model’s theoretical resistance to re-identification techniques” and “metrics on the likelihood of re-identification based on the current state-of-the-art.”

Because this assessment depends on a constantly evolving state of the art, the risk of identification can rarely be considered entirely absent. This may only be achievable when the original dataset is anonymous from the outset or has been effectively anonymized beforehand, although it may reduce model performance in some cases. For regulatory sandbox purposes, we therefore propose a precautionary approach. Whenever the dataset held at each local node is not anonymous, the parameter updates exchanged within the FL architecture should be treated as personal data even if the design phase tests indicate only a low level of risk.

For example, several hospitals could use FL to jointly train a model for predicting clinical deterioration. If the exchanged parameter updates are treated as personal data, their transmission, storage, aggregation, and access would also fall within the GDPR governance framework. During sandbox assessment, participating institutions could therefore define the purposes and responsibilities associated with these operations and assess appropriate safeguards before deployment.

### Ensure Traceable Access to Clinical Records by AI Agents in Health Care

The AI Act was originally conceived for comparatively static AI systems, yet recent years have seen the rapid emergence of AI agents. In this discussion, we use this term to refer to systems capable of executing multistep tasks, accessing external tools, and autonomously performing certain actions. These systems are becoming increasingly relevant in health care [32-34] and have shown potential performance gains over state-of-the-art models [35]. This evolution raises important regulatory challenges under Article 14 of the AI Act, which requires human oversight measures proportionate to the risks, level of autonomy, and context of use of high-risk systems. At the same time, the regulatory treatment of AI agents remains at a preliminary stage as current European Commission guidance acknowledges that the concept is still evolving and that the relevant regulatory considerations are not yet fully settled.

Building on the human oversight principles established in Article 14, we propose operational safeguards for testing AI agent access to clinical records within health care regulatory sandboxes. Each access should be recorded as an auditable event and linked to a defined purpose, an identifiable nonhuman identity, and a responsible human or organizational authority. For example, an AI agent used to coordinate follow-up care after hospital discharge could access appointment schedules, referral information, and pending test results. During sandbox testing, it could be verified that the agent accesses only the information required for this task, that every access is logged, and that responsibility remains attributable to a human supervisor. Access to unrelated records or unnecessary information should trigger review and accountability mechanisms equivalent to those triggered by unjustified access by human professionals.

The recommendations developed in the preceding sections are consolidated into a practical overview (Table 2) for the design and implementation of health care AI regulatory sandboxes.

**Table 2.** Principal regulatory and technical challenges of the European Union (EU) AI Act in health care and proposed measures for regulatory sandboxes.

EU AI Act limitations	Proposed recommendations
Model compression may reduce computational complexity without reducing clinical impact	Require developers to disclose the use of parameter compression techniques (including tensor-based architectures)
Lack of specific technical requirements for explainability	Select explainability approaches according to the clinical question and the characteristics of the datasets
Health care datasets may contain representation biases	Request dataset documentation and auditing to identify bias, particularly underrepresentation of clinically relevant groups, and justify unavoidable limitations
Federated learning parameter updates may allow information about patients to be inferred	Treat federated learning parameters as personal data whenever local datasets are not anonymous, even when the risk of reidentification is low
AI agents may access clinical records without sufficient traceability and accountability	Ensure that AI agent access to clinical records is logged as auditable events linked to a defined purpose and responsible authority

## Conclusions

The EU AI Act provides a general legal framework for the governance of AI in health care, but its practical effectiveness will depend on how its requirements are operationalized in concrete testing and deployment settings. In this regard, the recommendations proposed in this Viewpoint are intended to support the design of health care regulatory sandboxes that are more robust, transparent, and responsive to the specific challenges posed by medical AI systems. In particular, they address issues that are likely to become increasingly relevant in practice, including model compression, explainability, dataset bias, FL, and human oversight of AI agents. Taken together, these proposals aim to facilitate earlier risk identification, improve legal and technical clarity, and strengthen the supervised development of trustworthy AI in clinical contexts.

## Abbreviations

EU: European Union
FL: federated learning
FLOPS: floating point operations per second
GDPR: General Data Protection Regulation
